# Advanced Insights into Competitive Endogenous RNAs (ceRNAs) Regulated Pathogenic Mechanisms in Metastatic Triple-Negative Breast Cancer (mTNBC)

**DOI:** 10.3390/cancers16173057

**Published:** 2024-09-01

**Authors:** Amal Qattan, Taher Al-Tweigeri, Kausar Suleman, Wafa Alkhayal, Asma Tulbah

**Affiliations:** 1 Department of Molecular Oncology, King Faisal Specialist Hospital and Research Centre, Riyadh 11211, Saudi Arabia; 2 College of Medicine, Alfaisal University, Riyadh 11533, Saudi Arabia; 3 Department of Medical Oncology, Oncology Centre, King Faisal Specialist Hospital and Research Centre, Riyadh 11211, Saudi Arabia; ttwegieri@kfshrc.edu.sa (T.A.-T.); ksuleman@kfshrc.edu.sa (K.S.); 4 Department of Surgery, King Faisal Specialist Hospital and Research Centre, Riyadh 11211, Saudi Arabia; wkhayal@kfshrc.edu.sa; 5 Department of Pathology and Laboratory Medicine, King Faisal Specialist Hospital and Research Centre, Riyadh 11211, Saudi Arabia; tulbah@kfshrc.edu.sa

**Keywords:** triple-negative breast cancer (TNBC), metastasis, mTNBC, ceRNAs, ceRNETs, ceRNome, microRNAs, circRNAs, lncRNAs, biomarkers, therapeutic targets

## Abstract

**Simple Summary:**

Triple-negative breast cancer (TNBC) is difficult to treat. This is partly because each tumor is different and there are few drugs that are effective against this disease. Further, the high mortality from TNBC is primarily attributable to its spread beyond the primary tumor site, or metastasis. There has been a lack of understanding of common factors affecting metastasis in TNBC and, consequently, continued difficulty in effectively treating this deadly disease. A re-emerging concept regarding the broad regulation of factors affecting disease is that of competitive endogenous RNA networks. The recent study of these networks and how they regulate metastatic processes in TNBC is providing new insights into the predominant molecular factors and cellular signaling pathways involved in the progression of this disease. Here, we review this research and what it may mean for patients with metastatic TNBC (mTNBC) and advancements that may improve their treatment.

**Abstract:**

Triple-negative breast cancer is aggressive and challenging to treat because of a lack of targets and heterogeneity among tumors. A paramount factor in the mortality from breast cancer is metastasis, which is driven by genetic and phenotypic alterations that drive epithelial–mesenchymal transition, stemness, survival, migration and invasion. Many genetic and epigenetic mechanisms have been identified in triple-negative breast cancer that drive these metastatic phenotypes; however, this knowledge has not yet led to the development of effective drugs for metastatic triple-negative breast cancer (mTNBC). One that may not have received enough attention in the literature is post-translational regulation of broad sets of cancer-related genes through inhibitory microRNAs and the complex competitive endogenous RNA (ceRNA) regulatory networks they are influenced by. This field of study and the resulting knowledge regarding alterations in these networks is coming of age, enabling translation into clinical benefit for patients. Herein, we review metastatic triple-negative breast cancer (mTNBC), the role of ceRNA network regulation in metastasis (and therefore clinical outcomes), potential approaches for therapeutic exploitation of these alterations, knowledge gaps and future directions in the field.

## 1. Introduction to Metastatic TNBC (mTNBC)

Triple-negative breast cancer (TNBC) is an aggressive subtype lacking expression of epidermal growth factor receptor 2 (HER2), estrogen receptor (ER) and progesterone receptor (PR). TNBC accounts for 10–15% of breast cancer [1] and is characterized by high tumor grade, large tumor size, high mitotic rate and high rate of metastasis [2]. TNBC also presents challenges in treatment because of a lack of molecular targets and high molecular heterogeneity among patients [3]. In fact, response to conventional chemotherapy is limited to approximately 50% of TNBC patients [4]. Incomplete response to chemotherapy does not necessarily mean that a patient will progress to metastasis, but some may progress, potentially tying a lack of therapy response to the potential for metastasis. Estimates vary considerably, but actual metastatic rates are likely to be lower than 50%. There may also be survival mechanisms related to resistance to chemotherapy that also play a role in metastatic progression. Responses of metastatic triple-negative breast cancer (mTNBC) to chemotherapy are diminished in the 2nd line or higher [5]. Ultimately, mortality in TNBC patients is associated primarily with metastasis as the 5-year survival rate of localized TNBC is 91%, while that with distant metastasis is only 12% [1]. Compounding poor outcomes with the progression of TNBC, metastasis is also associated with the development of resistance to therapy in a disease that is already challenging to target therapeutically [6]. The mechanisms of metastatic progression of TNBC are varied and complex. One potential explanation for the common metastatic progression of TNBC despite the complexity of the process is the involvement of competitive endogenous RNA (ceRNA) regulatory networks affecting multiple metastasis factors. Global regulation of transcripts by microRNAs (miRNAs) is known to affect metastasis in TNBC [7]. As hand-in-hand regulators of miRNA activity, ceRNA networks are also involved in TNBC metastasis [8], potentially providing novel targets and markers that control progression-associated phenotypes globally or correlate with them. Specific examples of miRNAs and ceRNA networks affecting metastasis in TNBC are discussed herein.

### 1.1. The Metastatic Cascade in TNBC

Metastasis breast cancer is incurable and is the main cause of mortality in the majority of TNBC patients [9]. Metastatic TNBC commonly occurs in the brain and visceral organs [5]. The process is highly complex and occurs in multiple steps, including invasion, intravasation, circulation, extra-vasation and metastatic outgrowth (or colonisation), survival and proliferation in the invaded tissues [9]. In the initial steps of metastasis, epithelial-to-mesenchymal transition (EMT) is a critical hallmark that is related to the ability of tumor cells to migrate, invade and enter circulation [9]. Epithelial-to-mesenchymal transition (EMT) involves a distinct transcriptional program that was first characterized in embryonic development. This program includes transcripts that alter cytoskeletal remodeling, loss of cell–cell junctions and loss of polarity [10]. Orchestration of this program is influenced by non-coding RNAs, particularly miRs, which operate within feedback loops to control cellular phenotype [10]. Central transcriptional regulators of early EMT include ZEB1/2, Snail, Slug, and Twist, promoting migration and invasion during intravasation [9]. TGFβ-Smad signaling, a key driver of the epithelial-to-mesenchymal transition (EMT); TGF-β is a key player in initiating EMT by activating the Smad pathway, which involves the control of these transcription factors and the silencing of epithelial genes, such as E-cadherin and ZO-1, and promotes the expression of mesenchymal genes, such as vimentin and fibronectin [9,11]. However, the key molecular regulators that are truly relevant to TNBC metastasis in vivo remain elusive and controversial, as invasion, migration and intravasation have been suggested to rely on other factors, including chemokine signaling. Aside from their association with lethal disease progression, mesenchymal-like tumor cells can lie dormant and evade genotoxic killing of targeting proliferating cells but can escape dormancy and proliferate as metastatic tumors through transition back to epithelial phenotypes [9,12]. This dormancy and its duration may help explain why metastatic recurrence of breast cancer can occur on timescales ranging from months to decades. Research has suggested that conversion of TNBC tumor cells with a mesenchymal phenotype to those with an epithelial phenotype using retinoids in the neoadjuvant setting may improve treatment response and outcomes, spurring clinical trials [13]. Regardless of the complexity of EMT and metastasis mechanisms, a deeper understanding of how this deadly disease process is regulated is needed to develop targeted treatment for mTNBC.

### 1.2. Genetic and Phenotypic Features in TNBC

Aside from lacking HER2 and hormone receptor alterations, TNBC is associated with particular genetic and phenotypic alterations. Copy number variations are common in TNBC, and they typically affect alterations in multiple molecular pathways [14]. TNBC consists of subtypes that are delineated based on molecular and phenotypic features. Each subtype differs in prognosis and therapeutic approach. Lehmann et al. established a classification system in 2016 that divides TNBC in the following types: basal-like 1 (BL1), basal-like 2 (BL2), immunomodulatory (IM), mesenchymal (M), mesenchymal stem-like (MSL), and luminal androgen receptor (LAR) [15].

Basal-like breast cancer (BLBC) is characterized by high histological grade, proliferation and poor prognosis [9]. This subtype has been proposed to originate in luminal progenitor cells (LPCs) as opposed to normal differentiated breast tissue as previously hypothesized [16,17], and has unique genetic and phenotypic features with implications on therapy. This subtype is characterized by expression of cytokeratin (CKs), such as CK5/6, CK14, and CK17, cadherin and epidermal-like growth factor (EGFR) [18] and the common BRCA1/BRCA2 mutation [19,20]. While BRCA1/BRCA2 mutation is associated with all breast cancers [21], TNBC exhibits a higher proportion with BRCA1/2 mutation (20% with a germline mutation) than other breast cancer subtypes [21]. BRCA1/2 testing is now recommended for TNBC patients under 50 years of age [22]. Given that BRCA1/2 repress basal-like genes under normal physiologic conditions and are commonly mutated in basal-like breast cancer [21], this breast cancer subtype is representative of cancers with homologous recombination deficiency (HRD), which are more likely to be sensitive to poly adenosine diphosphate (ADP) ribose polymerase inhibitors (PARPi) [22]. Basal-like breast cancer also commonly harbors p53 mutation (>50%) and loss of Rb1 [23]. Even among basal-like breast cancers, there is heterogeneity. BL1 harbors alterations in cell cycle and DNA damage response genes, and BL2 expresses higher levels of growth factor signaling factors and myoepithelial markers [24,25].

Some molecular stratification systems include “claudin-low” TNBC tumors as a classification, which have features of stemness and EMT [23]. These tumors are enriched for genes with mesenchymal implications, including Snail, Twist and ZEB1 [23]. Accordingly, dormancy of tumor cells with this phenotype presents treatment challenges [12] and metastatic recurrence over highly variable durations [25]. In fact, decreased claudin1 expression in breast cancer has been found to correlate with poor prognosis and early recurrence of metastatic breast cancer (mBC) [26,27].

### 1.3. Immunogenic Potential in TNBC

Among breast cancer subtypes, TNBC has a relatively high mutational burden [28], although there is variability in mutational burden among TNBC tumors [14]. The tumor microenvironment of TNBC tumors also suggest immunologically “hot” status, with increased expression of vascular endothelial growth factor (VEGF), tumor-infiltrating lymphocytes (TIL) and tumor-associated macrophages (TAM) [29]. PD-L1 expression is relatively high in TNBC among breast cancer subtypes, suggesting again that it is highly immunogenic and implying potential for immunotherapy [30,31]. Despite the appearance of TNBC as ripe for immunologic control, clinical evidence suggests that immunotherapy has not reached its potential in the treatment of TNBC. PD-L1 inhibitors as single agents have been reported to only be effective in 20% of PD-L1-positive mTNBC [32]. Recently, trials with Atezolizumab or Pembrolizumab in combination with chemotherapy have shown promise in PD-L1-positive mTNBC; however, the FDA issued an alert following a trial showing no benefit in this population. As with traditional chemotherapy, response rates to immunotherapy or immunotherapy/chemotherapy combinations diminish in the 2nd line and higher.

Combinations of immunotherapy with non-chemotherapy drugs are also being investigated for the treatment of mTNBC. For example, trials for mTNBC have been conducted combining immunotherapy with the LIV-1 ADC ladiratuzumab vedotin, AKT inhibitors, anti-CD73, anti-VEGF/PD-1 bispecific antibodies and DNA vaccines, among others [32]. The targeting of STAT3, which is a player in TNBC proliferation and migration [33], may be another promising target in combination with immunotherapy, since it is dysregulated in both tumor and non-tumor cells in the tumor microenvironment [34]. Indeed, clinical investigations are underway for the STAT3 inhibitor ruxolitinib in combination with pembrolizumab in metastatic stage IV TNBC [34]. The potential effects of non-coding RNA regulation of these players in anti-tumor immunity in mTNBC are discussed further herein.

## 2. ceRNA Regulatory Networks (ceRNETs) in mTNBC

### 2.1. ceRNA Mechanisms

The term competitive endogenous RNA was coined in 2011 [35]. Conceptually, the ability of miRNAs to target transcripts is dependent not only on their own concentration, but that of competing miRNA response elements (MREs) found on other species of competing RNA. These MREs can be found in the 3′UTR of coding transcripts. Since 3′UTRs have variable length and expression in relation to coding sequences and can be expressed independently, they can significantly affect the competitive environment for miRNA regulation [36]. Other RNA species, including circular RNA (circRNA), long non-coding RNA (lncRNA), tRNA, ribosomal RNA (rRNA) and pseudogene RNA act as ceRNAs. Each RNA species discussed in the manuscript, including miRNAs, lncRNAs, tNRAs, rRNAs, circRNAs and pseudogenes, can act as ceRNAs within a regulatory network. Within a network, each acts in competition with the others for binding to MREs within mRNA transcripts. The sum of these entities is referred to as the “ceRnome”. Transcripts and other ceRNAs can regulate miRNAs in a reversal of the paradigm of miRNAs regulating transcripts. Multiple MREs can be shared, which compete for the binding of miRNAs, downregulating miRNA activity in an MRE-dose-dependent manner [35,37]. The transcription, degradation and association/dissociation rates of both the miRNA, miRNA targets determine the steady state [9]. Altered expression or activity of each affects the other, potentially amplifying the effect of the alteration through its interactions with the broader ceRNA regulatory network. This applies to tumorigenic alterations, such as that of expression or activity of transcription or signaling factors, causing more aggressive phenotypes than would be expected with alteration of the single miRNA or target alone. While there are few published databases dedicated to ceRNAs, a few do exist, including lncACTdb 3.0, RNAcentral, ceRDB, miRcode, DIANA tools, miRTarBase, LNCipedia, LncBook, and LncExpDB, which are searchable based on disease and specific RNA entities and provide links to original research publications.

### 2.2. Role of ceRNAs in TNBC Metastasis

**Figure 1:**
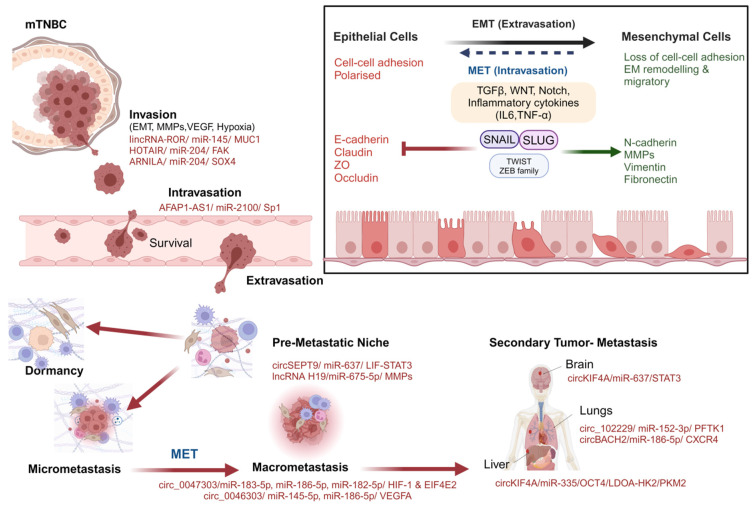
ceRNA programming in the metastatic cascade in breast cancer. The figure describes the stages of cancer metastasis and their associated ceRNA interactions. From Epithelial-to-Mesenchymal Transition to metastatic colonization, each stage involves different mechanisms for cancer cells to spread and establish in new tissues. Understanding ceRNA interactions is crucial for developing interventions to improve patient outcomes in metastatic cancer.

There is particular interest in the investigation of ceRNA networks in metastasis, as the majority of ceRNAs, such as lncRNAs, identified as having pathogenic roles in breast cancer have been found to do so through effects on EMT, migration, invasion and metastasis [38]. EMT and metastasis of breast cancer involve global reprogramming, which is affected broadly by ceRNA networks, as summarized in Figure 1 with specific examples.

#### 2.2.1. miRNAs in mTNBC

miRNAs affect metastasis of TNBC through modulation of epithelial to mesenchymal transition, invasion, migration and seeding in distant tissues [7]. Specific miRNAs are associated with metastasis to sites such as the brain, lungs and lymph nodes, making them potential markers for such distal spread [7]. Specific miRNAs have been found to affect tumor cellular phenotype through the regulation of epithelial and mesenchymal transcription/translation programs [10]. Banerjee et al. found that miRNAs hsa-miR-106b-5p, hsa-miR-148a-3p, hsa-miR-25-3p and hsa-let-7i-5p were common to non-coding RNA regulatory hubs associated with TNBC metastasis [39]. There are many other specific examples of miRNAs that are associated with or have functional roles in metastatic processes, some of which are summarized in Table 1. Some of the studies included in the table did not directly implicate or report a definitive miRNA competitor within the network, although the ceRNA entities listed are implied to have mechanistic links in competition with an miRNA or miRNAs. In these cases, no miRNA is listed.

#### 2.2.2. ceRNAs Regulating Migration and Invasion

Beyond miRNA regulation of transcripts, there have been many connections made between ceRNAs and disease progression and metastasis in TNBC. Several have functional implications for migration and invasion. For example, the lncRNA SNHG12 (small nucleolar RNA host gene 12) is upregulated in TNBC by c-Myc, where it promotes migration, invasion and metastasis to the lymph nodes, possibly through MMP13 (Matrix Metallopeptidase 13) de-repression [61]. The circRNA ciRS-7 also upregulates MMPs through competition with miR-1299 to increase migration and invasion of TNBC cells [43]. Commonalities in ceRNA regulation of MMPs and their role in migration, invasion and metastasis make this potential pathogenic mechanism interesting both mechanistically and clinically.

The lncRNA HOST2 (human ovarian cancer-specific transcript 2) promotes proliferation and migration by competing with let-7b to upregulate STAT3 [33], a potential target in both tumor cells and immune infiltrates, as discussed in the previous section. Likewise, circSEPT9 promotes LIF/STAT3-mediated migration and invasion through competition with miR-637, as shown in Table 1 [40]. These multiple independent findings regarding the role of ceRNA-regulated, STAT3-mediated effects on metastasis-relevant phenotypes warrant further investigation into this regulatory axis. It may be that ceRNA network regulation is a central mechanism controlling these phenotypes and that targeting these networks may be a powerful adjuvant approach to eliminate residual metastases. Such an approach against ceRNA networks rather than a single target may be more effective, for example, by targeting other mediators of invasion, migration and metastasis in addition to STAT3.

#### 2.2.3. ceRNAs Regulating EMT

**Figure 2:**
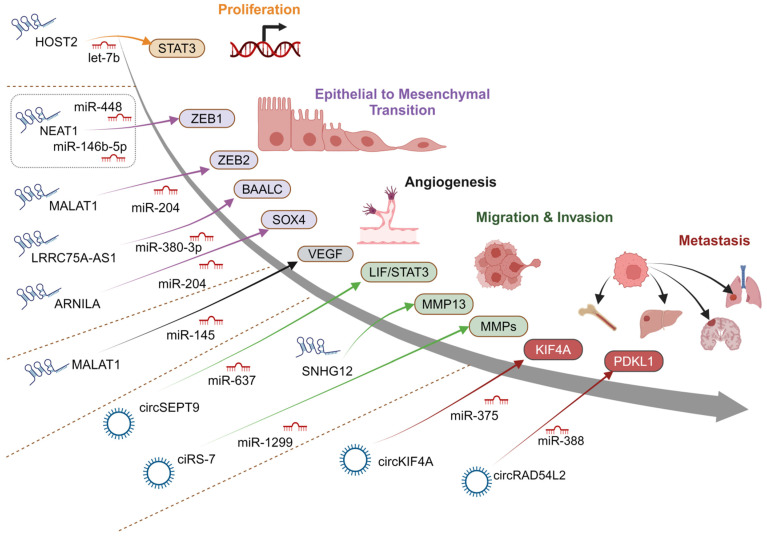
ceRNAs involved in cancer metastasis. ceRNAs regulation in mTNBC involves circRNAs and lncRNAs sponging miRNAs and thereby regulating the expression of target mRNAs to control EMT, stemness, proliferation, invasion, migration, and angiogenesis, contributing to different stages of cancer metastasis, as depicted in the figure.

Often, the migration and invasion of tumor cells go hand-in-hand with EMT, a key hallmark of metastasis, as shown in Figure 1. Several of the implicated ceRNA-mediated mechanisms promoting metastasis of breast cancer have evident effects on EMT (Figure 2). The lncRNA MALAT1 (metastasis-associated lung adenocarcinoma transcript 1) has been shown to regulate invasion, migration, EMT and metastasis in a variety of cancers, including TNBC, in which it correlates with poor prognosis [38]. It has been proposed that MALAT1 can promote EMT in breast cancer through ceRNA regulation of the PI3K/Akt pathway [65] and the miR-204/ZEB2 axis [66]. It has been found that lincRNA-ROR, which has altered expression in TNBC [68], promotes EMT, stemness, invasion and metastasis in TNBC cell lines [64]. Other ceRNAs that are associated with EMT in TNBC include HOTAIR (HOX transcript antisense intergenic RNA), HULC (Highly upregulated in liver cancer) and NEAT1 (Nuclear paraspeckle assembly transcript 1), all promoting a mesenchymal phenotype [68]. NEAT1 has been implicated in the proliferation, migration, EMT, stemness and metastasis via multiple mechanisms [69,70,71,72,73]. Modulation of miR-448 and resulting upregulation of ZEB1 have been identified as mechanisms of NEAT1-promoted breast cancer progression [73]. The targeting of miR-146b-5p in breast cancer tissues and cell lines has also been implicated in the role of NEAT1 in EMT, progression and survival in breast cancer [72]. HULC, which is associated with metastasis in TNBC [74], can influence tumor cells toward progression through competitive interaction with miR-200a-3p [68,75]. In breast cancer, the miR-200 family and p53 gene regulation represent an important axis that regulates EMT [67]. This axis controls mesenchymal transcription factors ZEB1 and ZEB2 and has been found to be influenced by a complex two-way feedback mechanism involving a network of ceRNAs [67]. The lncRNA LRRC75A-AS1 competes with miR-380-3p to upregulate the BAALC oncogene in breast cancer, resulting in increased invasion and EMT [54]. ARNILA lncRNA also promotes EMT, invasion and metastasis through miR-204 sponging and upregulation of Sox4 expression [52], as summarized in Figure 2.

#### 2.2.4. ceRNAs Regulating Stemness

Stem cell-like features in tumor cells, represented in a population referred to as cancer stem cells (CSCs), are proposed to be drivers of recurrence and chemoresistance [76]. This process is closely interrelated with EMT [77]. In fact EMT can directly promote stem cell programming [78]. In breast cancer cells, the 3′UTR of the STARD13 mRNA can act as a ceRNA that regulates stemness, EMT and metastasis [79,80]. The STARD13-associated ceRNA network can suppress stemness through inhibition of YAP/TAZ “stemness factors” activity and subsequent regulation of both Hippo and Rho-GTPase/F-Actin signaling [81]. STARD13 3′UTR was also found to inhibit migration and invasion in breast cancer cells by upregulation of TP53INP1, a suppressor of metastasis, through competition for miR-125b binding [79]. Given the relationship between EMT and stemness, other ceRNA networks known to regulate invasion, migration and EMT may also have deeper roles in the generation of CSCs and their ability to drive recurrence and chemoresistance, justifying further investigation into these potential pathogenic functions.

#### 2.2.5. ceRNAs Regulating the Immune Microenvironment

The immune microenvironment within specific tissues in which distant metastases occur can affect the ability of tumor cells to survive and proliferate in those tissues. As such, ceRNAs that regulate tissue-specific metastasis of breast cancer through modulation of anti-tumoral immunity have been discovered [82]. For example, loss of the lncRNA XIST (X-inactive specific transcript) can promote M2 reprogramming of microglial macrophages through release of exosomal miR-503, leading to the promotion of brain metastasis [83]. High expression of the nuclear lncRNA MIAT (Myocardial Infarction Associated Transcript) in breast tumor tissues is associated with increased infiltration of anti-tumor immune cells and improved response to immunotherapy [84]. However, detection of high MIAT expression in serum was found to be positively associated with clinical stage and metastasis in breast cancer [84,85]. Copy number amplification-associated high expression of LINC00467 (Long intergenic non-coding RNA 467) in breast cancer correlates with decreased immune and stromal infiltration and poor relapse-free survival and distant metastasis-free survival [86]. Conversely, the immune environment can regulate ceRNA networks. Interleukin (IL-6) can promote transcription of the novel lncRNA AU021063, which stabilizes Trib3 (tribbles homolog 3) to activate Mek/Erk signaling and promote breast cancer metastasis [87]. Additionally, expression of the lncRNA HISLA (HIF-1α stabilizing long non-coding RNA) in tumor-associated macrophages is associated with lymph node metastasis and shorter disease-free survival in breast cancer patients [82,88]. Given these lines of evidence, the immunologic role of ceRNA networks is of importance to the understanding of metastasis in breast cancer.

#### 2.2.6. circRNA Implications for Metastasis and Clinical Progression

Numerous circRNAs regulating metastasis and progression of breast cancer have been identified, including circBCBM1 (hsa_circ_0001944), promoting brain metastasis, and circIKBKB (hsa_circ_0084100), derived from the IKBKB gene (encoding inhibitor of NF-κB kinase subunit beta), activating the NF-κB pathway and promoting bone metastasis [8]. In TNBC, circTADA2A (circRNA transcriptional adaptor 2A) was found to be downregulated resulting in increased expression of SOCS3 (Suppressor of cytokine signaling 3) via competition with miR-203a [47]. This promotes aggressiveness and metastasis. Conversely, progression of TNBC is repressed by circFBXW7, which competes with miR-197-3p to upregulate FBXW7 and thereby inhibit migration of TNBC cells [48]. CircNR3C2 (has_circ_0071127) is downregulated in TNBC and inversely correlated with distant metastasis and poor survival/cancer lethality [44]. This non-coding RNA competes with miR-513a-3p, resulting in increased expression of the tumor suppressor HRD1 in TNBC. CircRAD54L2 is a competitor of miR-888, thereby mediating PDK1-dependent invasion and metastasis [46]. CircKIF4A promotes migration through miR-375 sponging and subsequent KIF4A upregulation, as shown in Table 1 [41].

Knowledge of the role of ceRNA networks in TNBC metastasis is relatively new. Delineation of truly metastasis-relevant ceRNA networks and transcriptional hubs of these networks is continuing and will be important for the utilization of this knowledge to provide mechanistic understanding and better treatment of mTNBC. CeRNAs that have been reported in the literature as having effects on TNBC progression and/or metastasis are listed in Table 1.

## 3. Clinical Implications and Therapeutic Targeting

There is a recent history of targeting EMT and stemness in cancer as a means of eliminating tumor cells with metastatic potential and chemoresistance. Agents targeting stemness-related factors include Notch inhibitors, STAT3 inhibitors, Wnt inhibitors, PI3K inhibitors, SMO antagonists, LRP5/6 inhibitors (low density lipoprotein receptor-related proteins 5 and 6), ROR1 inhibitors (receptor tyrosine kinase orphan receptor 1), YAP1 inhibitors and β-catenin regulators, among others [89]. Some of these agents have been investigated preclinically and clinically for some time with varied success.

**Figure 3:**
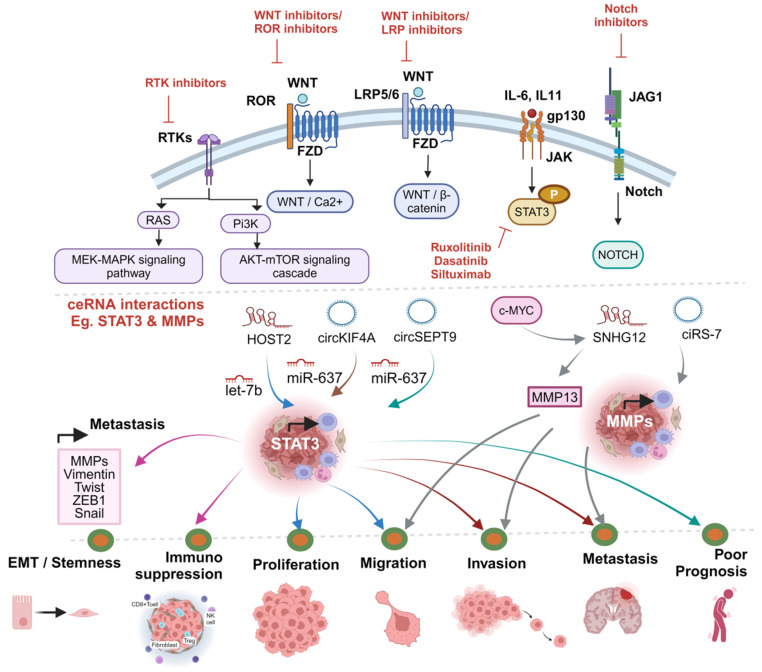
Clinical relevance of ceRNA interactions: therapeutic targets, like Notch, STAT3, Wnt, RTK, PI3K, and LRP5/6 inhibitors, are pursued. STAT3 and MMPs regulate EMT/stemness, immunosuppression, proliferation, migration, invasion, and metastasis. ceRNA interactions highlight key roles in these processes, emphasizing their significance in patient outcomes.While STAT3 is reported to control EMT/stemness related genes, like Vimentin, Twist, ZEB1, Snail and MMPs, thereby promoting metastasis, they are also known to contribute to immunosuppression. The upregulation of STAT3 related ceRNA interactions at various, like proliferation and migration (HOST2/let7b/STAT3), invasion and metastasis (circKIF4A/miR-637/STAT3) and poor prognosis (circSEPT9/miR-637/STAT3), are described in the figure with different arrow colors. Similarly the ceRNA interactions ciRS-7/MMPs and c-MYC induced SNHG12/MMP13 axis in promoting migration, invasion and metastasis of TNBC are highlighted in the figure.

As a strategy to more broadly target EMT and CSC programming, there is a rationale for targeting ceRNA regulatory pathways involved in these processes. A related ceRNA-regulated pathway that stands out in the literature is that of STAT3. STAT3 has been directly targeted for cancer therapy. Targeted mechanisms of STAT3 involvement in cancer pathology include immunosuppression [34] and promotion of stemness [90]. Targeting of the latter mechanism and its modulation using STAT3 inhibition has been demonstrated in breast cancer stem-like cells [90]. Target genes of STAT3 include those encoding invasion-related matrix metalloproteinases (MMPs) and EMT-related genes, including Vimentin, Twist and ZEB1 [91]. Inhibition of the STAT3 pathway has the potential to prevent tumor progression, treat residual disease and promote anti-tumor immunity. Several small molecule inhibitors of STAT3 exist, a few of which have been used in trials [91,92]. Indirect inhibitors of the STAT3 pathway, including Ruxolitinib, Dasatinib and Siltuximab, have been FDA approved [34]. Several ceRNAs that regulate STAT3 have been identified in TNBC, as summarized in Figure 3.

The lncRNA HOST2 upregulates STAT3 through competition with let-7b, promoting proliferation and migration in TNBC [33] The circRNA circKIF4A (circ_0007255) was recently shown to upregulate STAT3 by competing with miR-637, thereby promoting brain metastasis in TNBC [42]. CircKIF4A expression was found to be increased in metastases of breast cancer and in breast cancer cell lines. Inhibition of this ceRNA decreased invasion and brain metastasis in TNBC cell lines. Another circRNA, circSEPT9, has been shown to associate with advanced clinical stage and poor survival by sponging miR-637 and upregulating LIF/STAT3 signaling, as demonstrated in Table 1 and Figure 2 [40]. Knockdown of circSEPT9 resulted in decreased proliferation, migration and invasion in TNBC cells in vitro and tumor growth and metastasis in vivo.

MMPs are also promising targets of therapy that facilitate multiple steps in the metastasis cascade, including, migration, invasion, intra- and extra-vasation, neutralization of anti-tumor immunity and angiogenesis [93]. Further, expression of MMPs impacts cancer prognosis as a function of metastatic potential [94]. CeRNAs that modulate tumor progression phenotypes through regulation of MMPs are compelling candidate entries into targeted blockade of cancer-related MMPs in TNBC. Transcriptional activation of lncRNA SNHG12 downstream of c-Myc can upregulate MMP13 and promote proliferation and migration in TNBC cells [61]. The circRNA ciRS-7 facilitates the maintenance of metastatic phenotypes in TNBC cells, including invasion and migration, through competitive interaction with miR-1299 and subsequent upregulation of the expression of MMPs [43].

Another potentially targetable mechanism of metastasis in TNBC is the miR-200 family/p53/ZEB1/2 axis. This EMT-controlling axis is regulated by several ceRNAs, as outlined in Section 2.2.3, including MALAT1, circ-ZEB1, HULC, NEAT1 and lincRNA-ROR [64,66,73,75,95]. These ceRNAs may represent novel targets for the comprehensive inhibition of metastatic tumors in TNBC.

## 4. Future Perspectives: Challenges and Research Directions for the Clinic

As we move toward a more complete understanding of ceRNA network regulation as it relates to both physiology and disease, a better grasp of ceRNA hierarchies involved in cross-regulation within networks and the balance and stoichiometry of ceRNA/miRNA interactions will be required. This stoichiometry is complex, as it also relies on the numbers of miRNA responsive elements within ceRNAs. Experimental and mathematical models have been applied to these questions, although conclusions have been variable and contrasting [96]. Technical advances in experimental tools and methods of detection and quantification of ceRNAs and the ability to interpret the resulting data are also critical. To that end, computational approaches are being introduced to make ceRNA research more efficient [97]. Given the application of antisense oligonucleotides in targeting miRNAs and ceRNAs, effective delivery of RNA agents to target tissues also presents a current challenge. These challenges must be met to realize the therapeutic potential of ceRNA network targeting.

While there have been advances in the treatment of metastatic TNBC, including research into immunotherapy, PARP inhibition with immunotherapy or as maintenance after platinum, the targeting of PI3K, PKB (protein kinase B (PKB), also known as Akt) or mTOR and antibody–drug conjugates, chemotherapy remains central to its treatment, and advanced therapies have not yet significantly made impacts on overall survival [5]. Given the need for more effective therapy in terms of overall survival, the control of metastasis through the targeting of central regulators of EMT and stem-like phenotypes is an attractive approach. CeRNAs may present that opportunity.

The targeting of STAT3 in combination with immunotherapy has become an approach of interest. The simultaneous regulation of anti-tumor immunity and stemness by STAT3 could present a powerful vulnerability in metastatic cancer, bringing attention to the involvement of several ceRNAs in the STAT3 signaling pathway, as discussed above. The targeting of ceRNA networks that regulate the EMT-central axis around p53/mir-200 family/ZEB1/2 is another potential means of controlling metastasis of TNBC. Indeed, agents that inhibit the related ceRNAs NEAT and MALAT1 have been demonstrated in experimental models to improve drug resistance and tumor cell survival, respectively [98]. MMPs are a validated drug targets in cancer [99] that are also a recurring theme in ceRNA regulation in TNBC, e.g., SNHG12 regulation of proliferation, migration and apoptosis through MMP13 regulation [61] and ciRS-7 maintenance of metastatic phenotypes through MMP regulation [43]. These mechanisms represent potentially exploitable clinical targets informed by current and developing knowledge of ceRNA networks.

In addition to research into therapeutic targeting of ceRNAs, the detection of ceRNAs in circulation as diagnostic or prognostic markers is being investigated [38]. High levels of circulating lncRNA HOTAIR has been found to correlate with poor drug response and poor prognosis in breast cancer [100]. Circulating lncRNA MIAT may also potentially serve as a prognostic marker in TNBC, given its correlation with clinical stage and metastatic progression [85].

## 5. Conclusions

A compelling approach to cancer treatment is to focus on control of disease progression. Indeed, it is metastasis that most often dictates survival outcomes in TNBC. Mechanisms involved in the metastatic cascade are becoming clearer as reprogramming toward EMT and stemness come to the forefront. It is not an easy task to determine how these complex transcriptional programs are regulated and can be manipulated therapeutically. However, we are now revealing evidence that ceRNA networks are central to the regulation of these programs, presenting an unprecedented opportunity to exploit hubs of broad transcriptional regulation to control metastatic phenotypes. Prime examples in TNBC metastasis include ceRNA networks regulating the p53/miR-200 family/ZEB1/2 axis and those regulating STAT3 signaling. Specific changes in ceRNA regulators can affect the propensity of tumors to metastasize to specific tissues, indicating that different transcriptional programs may be required for tissue-specific metastasis. As these programs and their central regulators come into view, we are compelled to leverage that knowledge for the effective treatment of metastatic disease.

## Figures and Tables

**Table 1:** ceRNAs affecting progression/metastasis TNBC.

Type	ceRNA	Genomic (Chr.) Location for ceRNA	miRNAs	Target Transcripts	Physiologic/ Pathologic Functions	PubMed ID	Reference
circRNA	circSEPT9	17q25.3	miR-637	LIF/STAT3	LIF/STAT3 Signaling, Migration, Invasion, Proliferation	32264877	Zheng 2020 [40]
circRNA	circKIF4A	Xq13.1	miR-375	KIF4A	Proliferation and Migration	30744636	Tang et al., 2019 [41]
circRNA	circKIF4A	Xq13.1	miR-637	STAT3	Brain Metastasis	38029538	Wu et al., 2024 [42]
circRNA	ciRS-7	Xq27.1	miR-1299	MMPs	Migration and Invasion	30072582	Sang et al., 2018 [43]
circRNA	circNR3C2	4q31.23	miR-513a-3p	HRD1	Proliferation, Migration, Invasion, EMT	33530981	Fan 2021 [44]
circRNA	circAHNAK1	11q24.3	miR-421	RASA1	Inhibits Proliferation and Metastasis	31857500	Xiao et al., 2019 [45]
circRNA	circRAD54L2	17q11.2	miR-888	PDK1	Invasion, Metastasis, Proliferation	36334805	He et al., 2023 [46]
circRNA	circTADA2A-E6	17p13.1	miR-203a	SOCS3	Migration, Invasion	30787278	Xu et al., 2019 [47]
circRNA	circFBXW7	4q31.3	miR-197-3p	FBXW7	Migration, Proliferation	31536884	Ye et al., 2019 [48]
circRNA	hsa_circ_102229	Xq23	miR-152-3p	PFTK1	Tumorigenesis, Lung Metastasis	34031947	Du et al., 2021 [49]
LncRNA	BORG	6p21.1		NF-kB	Doxorubicin resistance, Metastasis	30467380	Gooding et al., 2019 [50]
LncRNA	SOX2-OT	3q26.3	miR-942-5p	PIK3CA	Activates Pi3k/Akt, activates metastasis	34997317	Zhang et al., 2022 [51]
LncRNA	ARNILA	12q23.1	miR-204	SOX4	EMT, invasion, Metastasis	29844570	Yang et al., 2018 [52]
LncRNA	ST8S1A6-AS1	5q21.3	miR-145-5p	CDCA3, p53/p21	Proliferation, Metastasis	35730485	Qiao et al., 2022 [53]
LncRNA	LRRC75A-AS1	2q31.2	miR-380-3p	BAALC	Proliferation, Invasion, EMT	32811810	Li et al., 2020 [54]
LncRNA	LncRNA SNHG6	8q13.3	miR-125b-5p	BMPR1B	Proliferation, Migration, Apoptosis	34026654	Lv et al., 2021 [55]
LncRNA	LRP11-AS1	6q22.31	miR-149-3p	NRP2	Tumorigenesis, Metastasis	35279146	Li et al., 2022 [56]
LncRNA	lincRNA-ROR	18q21.31	miR-145	MUC1	Invasion, Metastasis	29673594	Ma et al., 2018 [57]
LncRNA	DANCR	4q12	miR-874-3p	TUFT1	Invasion	33058834	Wu et al., 2020 [58]
LncRNA	HOTAIR	12q13.13	miR-146a-5p		Lymph Node Metastasis, LAR Subtype	31205562 31672084	Collina 2019 [59], Liang 2019 [60]
LncRNA	HOST2	Xq28	let-7b	STAT3	Proliferation, Migration	32248842	Hua et al., 2020 [33]
LncRNA	SNHG12	17q25.3		MMP13	Proliferation, Migration, Apoptosis	28337281	Wang et al., 2017 [61]
LncRNA	SENP3-EIF4A1	12q24.31	miR-195-5p	EIF4A1/CCNE1	Progression	33791304	Chen et al., 2021 [62]
LncRNA	HNF1A-AS1	12q24.31	miR-32-5p	RNF38	Progression	33603481	Yang et al., 2021 [63]
LncRNA	LincRNA-ROR	18q21.31	miR-145		EMT, Invasion, Metastasis, Stemness	24922071	Hou et al., 2014 [64]
LncRNA	MALAT1	11q13.1	miR-201	PI3K/Akt, ZEB2	EMT, Invasion, Migration, Metastasis	25431257 28675122	Dong 2015, [65], Wang 2017 [66]
miRNA	miR-200 family		miR-200 family	p53, EMT-TFs such as ZEB1/2	EMT, Metastasis	33414456	Parfenyev et al., 2022 [67]

## Data Availability

Not applicable.
